# Sonoporation generates downstream cellular impact after membrane resealing

**DOI:** 10.1038/s41598-021-84341-3

**Published:** 2021-03-04

**Authors:** Xinxing Duan, Qian Zhou, Jennifer M. F. Wan, Alfred C. H. Yu

**Affiliations:** 1 Schlegel Research Institute for Aging & Department of Electrical and Computer Engineering, University of Waterloo, Waterloo, ON Canada; 2 School of Biological Sciences, The University of Hong Kong, Hong Kong, China

**Keywords:** Biomedical engineering, Biological physics, Drug delivery, Cell biology

## Abstract

Sonoporation via microbubble-mediated ultrasound exposure has shown potential in drug and gene delivery. However, there is a general lack of mechanistic knowledge on sonoporation-induced cellular impact after membrane resealing, and this issue has made it challenging to apply sonoporation efficiently in practice. Here, we present new evidence on how sonoporation, without endangering immediate cell viability, may disrupt downstream cellular hemostasis in ways that are distinguished from the bioeffects observed in other sonicated and unsonoporated cells. Sonoporation was realized on HL-60 leukemia cells by delivering pulsed ultrasound (1 MHz frequency, 0.50 MPa peak negative pressure; 10% duty cycle; 30 s exposure period; 29.1 J/cm^2^ acoustic energy density) in the presence of lipid-shelled microbubbles (1:1 cell-to-bubble ratio). Results showed that 54.6% of sonoporated cells, despite remaining initially viable, underwent apoptosis or necrosis at 24 h after sonoporation. Anti-proliferation behavior was also observed in sonoporated cells as their subpopulation size was reduced by 43.8% over 24 h. Preceding these cytotoxic events, the percentages of sonoporated cells in different cell cycle phases were found to be altered by 12 h after exposure. As well, for sonoporated cells, their expressions of cytoprotective genes in the heat shock protein-70 (HSP-70) family were upregulated by at least 4.1 fold at 3 h after exposure. Taken altogether, these findings indicate that sonoporated cells attempted to restore homeostasis after membrane resealing, but many of them ultimately failed to recover. Such mechanistic knowledge should be taken into account to devise more efficient sonoporation-mediated therapeutic protocols.

## Introduction

Sonoporation has been well-regarded as an emerging membrane perforation paradigm in cancer therapeutics^1, 2^ and drug/gene delivery^3–5^. This membrane perforation paradigm often works by applying low-intensity ultrasound to microbubble situated near the cell membrane to create transient pores that in turn serve as physical windows for extracellular molecules to infiltrate target cells without inducing undesired thermal effects^6^. From a drug delivery standpoint, one major advantage of sonoporation is its high spatiotemporal specificity^7^. Accordingly, it has strong potential in facilitating precise, controlled release of drug payload to target cells.

Alas, sonoporation-mediated drug delivery has yet to reach widespread clinical use, as the efficiency of this approach has been deemed to be mediocre^8^. Detailed understanding on sonoporation mechanisms has long been regarded as vital to determine how sonoporation may be efficiently realized without inducing unwanted confounding effects^9, 10^. On this topic, not only is it important to unravel the acute biophysical impact of sonoporation^11–15^, the downstream cellular bioeffects of sonoporation after membrane resealing also need to be accounted for^16^. Without such mechanistic understanding, pursuing rational use of sonoporation in biomedicine inherently becomes more challenging^8, 10, 16^.

Although it is recognized that analyzing the downstream bioeffects of sonoporation is critical to enhance its practical efficiency, such analyses are not trivial to carry out properly from an experimental design standpoint. One known issue is that, when using ultrasound and microbubbles to induce sonoporation in a cell population, such exposure would lead to a heterogeneity of response among the sonicated cells^17, 18^. In particular, not all sonicated cells may undergo sonoporation, and some of them may instantly lose viability^17, 19^. Such heterogeneity, if neglected, fundamentally represents a significant confounding factor in the analysis of sonoporation-induced cellular bioeffects. Previously, without acknowledging the heterogeneity of cell response, some studies have shown that ultrasound exposure in the presence of microbubbles may lead to cell lysis^20, 21^, apoptosis^22^, cell cycle arrest^23^, and morphological changes^24, 25^. Yet, it remains unclear as to whether these biological effects are specific to sonoporation or whether they are merely attributed to ultrasound exposure.

In this paper, we present a new substantiated body of evidence on the downstream cellular impact of sonoporation by properly differentiating the bioeffects of viable sonoporated cells from other sonicated cells. The focus of our investigation is on analyzing sonoporated cells’ post-resealing viability, proliferation trend, cell-cycle distribution, and heat shock protein-70 (HSP-70) expression level, the last of which is known to be upregulated when cells are stressed^26^. Such an analysis allows us to more precisely identify whether, after membrane resealing has taken place, sonoporation may induce cellular impact that could be cytoprotective or cytotoxic in nature. Here, we have sought to test the overall hypothesis that various downstream bioeffects of sonoporated cells, such as proliferation behavior and HSP-70 expression levels, are different from those for cells that merely received ultrasound exposure. We further hypothesized that, in sonoporated cells, alterations in cell proliferation kinetics and HSP-70 expressions may emerge hours after ultrasound exposure when membrane integrity has long been restored.

## Results

**Figure 1:**
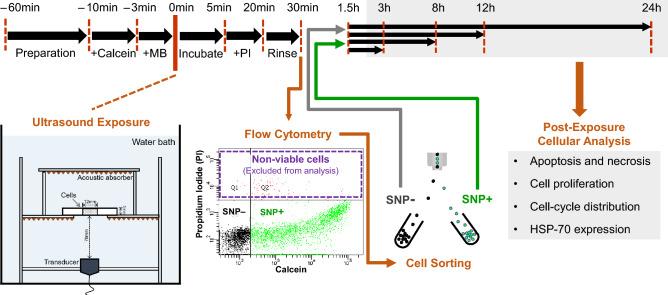
Experimental protocol for investigating the downstream cellular impact of sonoporation. A calibrated acoustic exposure platform was used to deliver ultrasound pulses to cell samples at 0 min. Fluorescence activated cell sorting (FACS) was then performed to isolate sonoporated cells (SNP+ group) and unsonoporated cells (SNP– group). The development trends of each sorted cell group were then assayed at different post-exposure time points over a 24 h period.

A broad range of sonoporation-induced cellular impact was unraveled using: (i) a meticulously designed experimental protocol for population-based sonoporation bioeffect studies^27^; (ii) multiple bioassays that gauged the time-lapsed viability, proliferation, cell-cycle behavior, and HSP-70 expression of sonoporated cells. As illustrated in Fig. 1 and described in the Methods section, the protocol involves the use of a calibrated, immersion-based ultrasound exposure setup (1 MHz ultrasound frequency, 10% duty cycle, 0.50 MPa in-situ peak negative pressure, 30 s exposure period, 29.1 J/cm^2^ acoustic energy density). Experiments were conducted using HL-60 leukemia cells (CCL240, American Type Culture Collection, Manassas, VA, USA) as the cell model, because their pro-apoptotic protein deficiency^28^ suitably acted as an experimental control in our investigation that included analysis of sonoporation-induced anti-proliferation effects^29^. Microbubbles (USphere Prime; Trust Biosonics, Hsinchu, Taiwan) were injected into the cell chamber at a 1:1 cell-to-bubble ratio to serve as sonoporation agents. Such a cell-to-bubble ratio is known to efficiently trigger the onset of sonoporation without significantly inducing instant cell death^20^. As shown previously, our exposure protocol yielded an average sonoporation rate of 41.9% among sonicated HL-60 cells^27^.

In this investigation, sonicated cells were re-incubated for 5 min after exposure to allow for membrane resealing to take place. Such post-exposure re-incubation period effectively took into consideration previous direct observations that showed how membrane resealing would typically happen within 1 min from the onset of a resealable sonoporation episode^11^. Afterward, fluorescence activated cell sorting (FACS) was carried out. Two subpopulations were accordingly isolated for cellular impact analysis: (i) viable sonoporated cells (SNP+ group); (ii) viable unsonoporated cells (SNP– group). To facilitate comparison, experiments were also conducted for: (i) a control cell group (CTL group) with sham exposure; (ii) a sonicated cell group (US group) that received exposure in the absence of microbubbles. As presented in the following subsections, our investigation has yielded a new, unique body of experimental insights on the downstream bioeffects that emerged in viable sonoporated cells (i.e. SNP+ group) at different post-resealing time points.

### Apoptosis and necrosis observed in sonoporated cells

**Figure 2:**
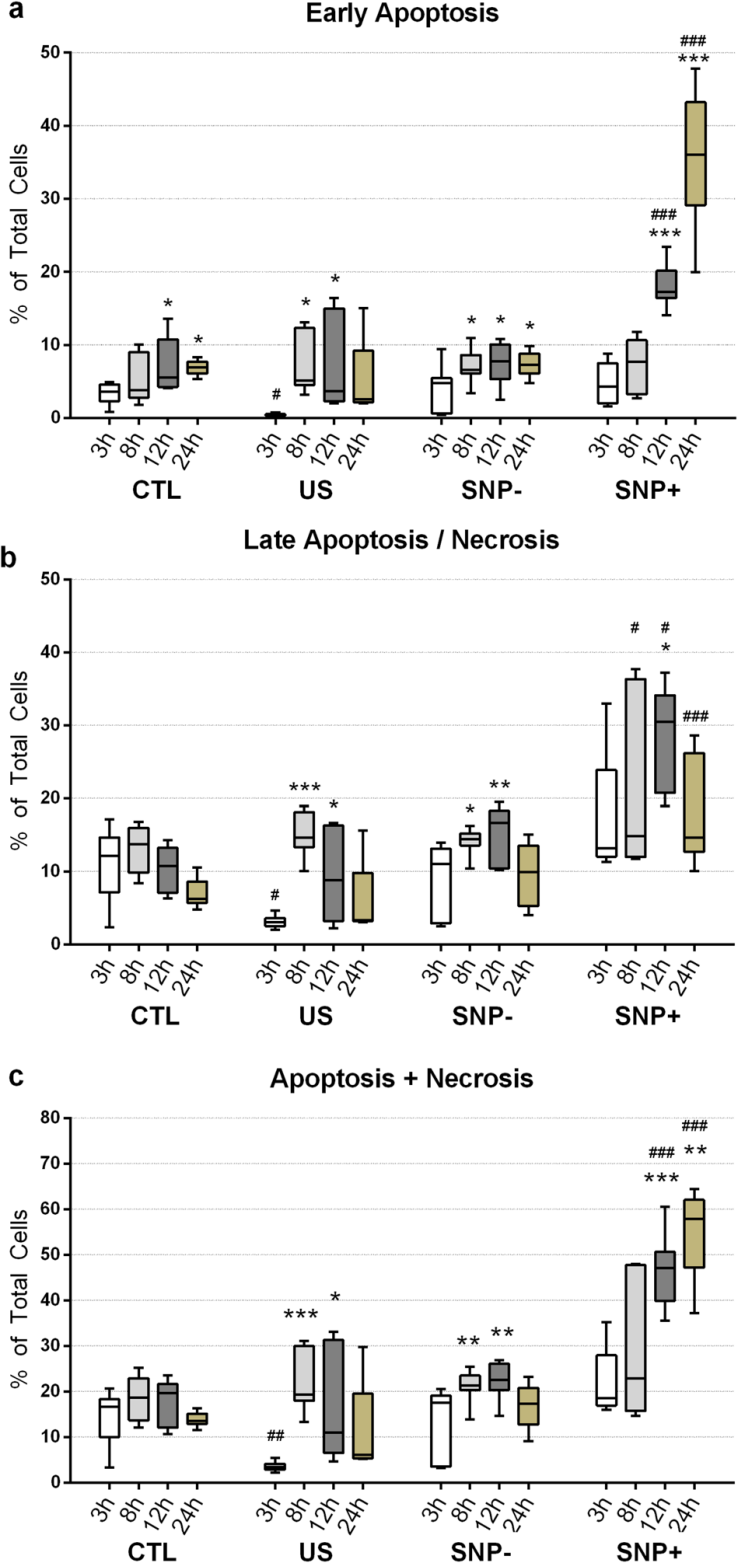
Cytotoxicity trends at different post-exposure time points (3 h to 24 h) for sonoporated (SNP+) and unsonoporated cells (SNP–) that were initially viable after exposure. Each box-whisker plot shows, for each sorted cell group, the percentage of cells in **(a)** early apoptosis, **(b)** late apoptosis or necrosis (i.e. dead), and **(c)** either death pathway (i.e. sum of apoptosis and necrosis). Results are also shown for sham exposure cells (CTL) and cells that were sonicated in the absence of microbubbles (US) (*N *= 9; *,**, and *** respectively denote *p*<0.05, *p*<0.01, and *p*<0.001 versus the 3 h time point of the same group. # and ### respectively denote *p*<0.05 and p<0.001 versus CTL at the corresponding time point).

Cells in the SNP+ and SNP– groups showed different trends for early apoptosis over a 24 h post-exposure period, as determined using annexin-V/propidium iodide (PI) bivariate flow cytometry (Fig. 2a). The early apoptotic population of the SNP+ group progressively increased from an average of 4.6% at 3 h after exposure to 36.1% at 24 h after exposure. At 12 h and 24 h, the percentage of the SNP+ group’s early apoptotic cells were significantly higher than those at earlier time points (*p* < 0.001). The other three cell groups (CTL, US, SNP–) showed relatively low cases of early apoptosis over 24 h. Note that the US and SNP– groups exhibited a minor increase in early apoptosis at 8 h (*p* < 0.05) but observed no further increase thereafter. At 24 h, the early apoptotic population percentages of the US and SNP– groups were respectively found to be 5.3% and 7.4% on average. At this post-exposure time point, no statistically significant differences were found between US and SNP–, between US and CTL, and between SNP– and CTL.

For the SNP+ group, significant emergence of dead cells, which may stem from late apoptosis or necrosis, was found over 24 h. Figure 2b shows the trend of the percentage of dead cells in the four tested cell groups over 24 h after exposure. As can been observed, dead cells in the SNP+ group had a significantly higher population than those in the CTL group at 8 h (*p* < 0.05), 12 h (*p* < 0.001), and 24 h (*p* < 0.001). The average percentage of dead cells in the SNP+ group reached 28.8% at 12 h. The dead cell percentage subsequently dropped to 18.5% at 24 h after exposure because some dead cells, whether they originate from the apoptosis or necrosis pathways, had already been lysed by that time and could no longer be detected by flow cytometry. In contrast, the SNP– and US groups had dead cell percentages below 20% over 24 h. Both groups showed no significant difference in dead cells between them and with respect to the CTL group at 24 h.

The percentage of apoptotic and necrotic cells in each group at different post-exposure time points is plotted in Fig. 2c. The data points were derived by summing the early apoptotic (Annexin-V + and PI–) and dead cell (PI +) populations. The primary observation to be noted is that for cells in the SNP+ group, over half of them failed to survive at 24 h after exposure (apoptotic and necrotic cells on average accounted for 54.6% of group population). Since some dead cells were already lysed and were thus not detectable after 24 h, the actual number of apoptotic and necrotic cells in the SNP+ group may be even higher than that reported in Fig. 2c. Note that the significant rise in the SNP+ group’s apoptotic and necrotic cell subpopulation (*p* < 0.001) started to emerge at 12 h after exposure. In contrast, the average apoptotic and necrotic cell percentage in the SNP– group merely comprised 16.8% of the group population at 24 h after exposure. This trend is similar to that observed for the CTL and US groups.

### Viable sonoporated cells exhibited anti-proliferation behavior

**Figure 3:**
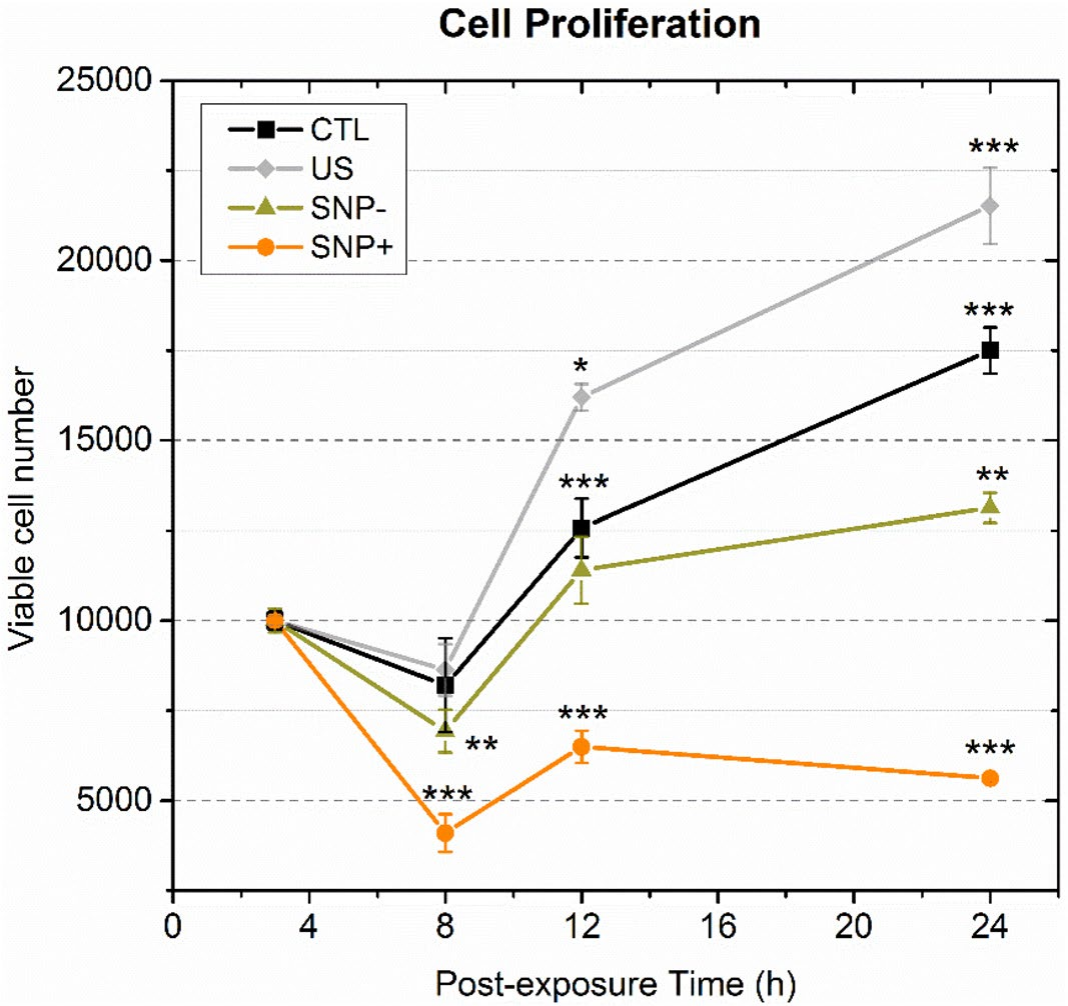
Growth curves of four sorted cell groups (SNP+, SNP–, US, CTL) over 24 h after exposure. Each line plots the total number of viable cells in a sorted cell group at 3 h, 8 h, 12 h, and 24 h after exposure. to evaluate Cell count was normalized to 10^4^ cells at the initial time point. Each data point, acquired from three experiments (*N*=3) with 3 to 5 population samples each, was presented as mean ± standard deviation (*, **, and *** respectively denote *p *< 0.05, *p *< 0.01, *p *< 0.001 versus the 3 h time point of the same group).

Proliferation of cells in the SNP+ group was found to be halted over a 24-h post-exposure observation period. Figure 3 presents the normalized cell population of each group at 3, 8, 12 and 24 h after exposure as obtained from a Cell Counting Kit-8 (CCK-8) bioassay. CTL, US and SNP– groups showed a rising trend in their population over 24 h, while the SNP+ group exhibited a substantial decrease in population. The population of CTL and US groups did not exhibit a statistically significant change from 3 to 8 h after exposure, whereas SNP+ and SNP– populations were decreased over the same period (*p* < 0.001 for SNP+ ; *p* < 0.01 for SNP–). Subsequently, at 12 h after exposure, the SNP– group by-and-large restored its population to the baseline level (with no statistically significant difference in comparison to the SNP– population at 3 h). In contrast, the SNP+ cells were still significantly lower than the initial population (*p* < 0.001). The low population of SNP+ persisted at 24 h after exposure, while the other three groups exhibited a population growth trend from 12 to 24 h.

To quantify the differences in the proliferation trends of the four groups, we calculated the post-exposure proliferation rate as the ratio of the population at 24 h to the population at 3 h. As shown in Table 1, the average proliferation rate of the CTL, US and SNP– cell groups were accordingly found to be 1.75, 2.12, and 1.31 respectively. In contrast, the average proliferation rate of the SNP+ group was estimated to be 0.56, which corresponds to an average population loss of 44% from 3 to 24 h. The population changes from 8 to 12 h (Δ_1_) and from 12 to 24 h (Δ_2_) were also calculated to evaluate the transient cell growth trend of each group. Results showed that over the period between 8 and 12 h, the SNP+ group population increased transiently by 60.1% on average (*p* < 0.001). Yet, this group’s population did not continue its rising trend from 12 to 24 h, as no statistically significant difference was found between the SNP+ group populations at 12 h and 24 h (the 13.2% decrease stated in Table 1 only exhibited weak statistical difference; *p* < 0.1). This population trend is different from the growth trends for the CTL, US, and SNP– cell groups, all of which experienced a statistically significant population increase from 8 to 12 h and from 12 to 24 h.

**Table 1:** Assessment of proliferation rates of the four sorted cell groups.

Group	Exposure			Average proliferation rate	Δ_1_ (%)	Δ_2_ (%)
	US	MB	SNP	3–24 h	8–12 h	12–24 h
CTL	−	−	−	1.75	55.5 ± 22.9	39.8 ± 13.2
US	+	−	−	2.12	88.7 ± 19.7	32.9 ± 6.6
SNP−	+	+	−	1.31	66.1 ± 26.9	15.5 ± 8.1
SNP+	+	+	+	0.56	60.1 ± 22.7	− 13.2 ± 4.7

### Time-lapsed disruption of cell cycle distribution after ultrasound exposure in presence of microbubbles

The results of cell proliferation rates suggested differences in the cell division activities of the four groups of cells. To investigate the impact of the exposure on each phase of cell division, a time-lapse cell cycle analysis was conducted at 3 h, 8 h, 12 h and 24 h after exposure via flow cytometry analysis of the cells’ deoxyribonucleic acid (DNA) contents. This analysis focused on the subgroup size of three cell-cycle phases: (i) G_0_/G_1_ phase (cells in quiescence or in preparation for DNA synthesis); (ii) S phase (cells undergoing DNA synthesis); (iii) G_2_/M phase (cells after completion of DNA synthesis but have not yet divided).

**Figure 4:**
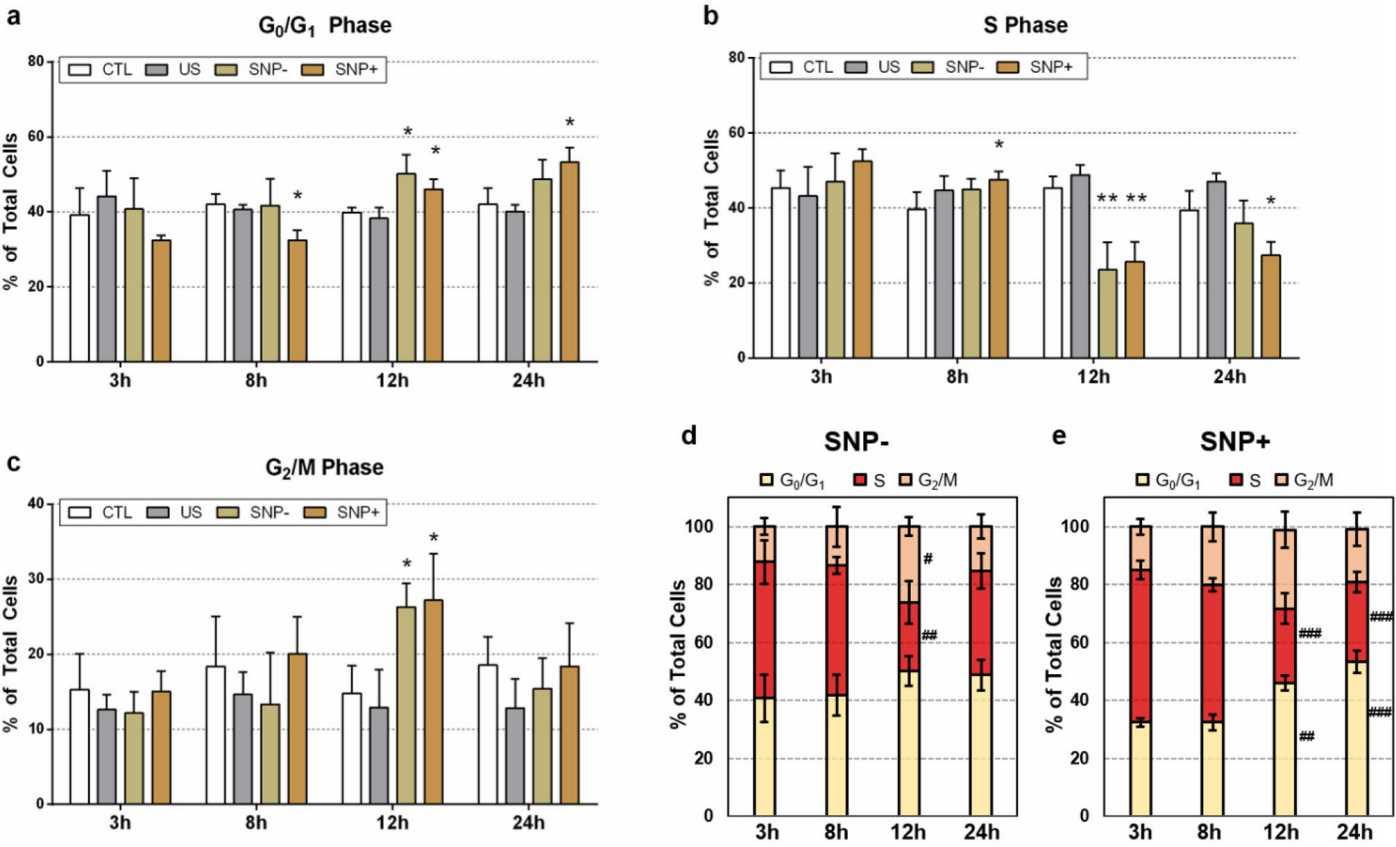
Time-lapsed cell cycle progression trends of four sorted cell groups (SNP+, SNP–, US, CTL) over a 24 h period. Percentages of cells in the following cell-cycle phases are shown at 3 h, 8 h, 12 h, and 24 h after exposure: **(a)** gap 0 or gap 1 (G_0_/G_1_); **(b)** synthesis (S); **(c)** gap 2 or mitosis (G_2_/M). Cell cycle distribution at the four post-exposure time points are also plotted as stacked bar charts for **(d)** SNP– and **(e)** SNP+ cell groups. Population percentages were calculated based on the total number of viable cells. Results were presented as mean ± standard deviation. Data points were acquired from three experiments (*N *= 3), each of which had 3 to 5 samples (*, **, and *** respectively denote *p *< 0.05, *p *< 0.01, and *p *< 0.001 versus CTL at the same time point. #, ##, and ### denote *p *< 0.05, *p *< 0.01, *p *< 0.001 versus the 3 h time point of the same group).

For the four cell groups of interest, Fig. 4a–c show the percentage of cells in the three phases as a function of post-exposure time. The CTL and US groups remained at similar distribution levels for each phase of cell cycle progression over 24 h after exposure. In contrast, the SNP+ and SNP– groups had shown fluctuations in the cell-cycle distribution from 8 h onward. Specifically, as highlighted in Fig. 4e, the percentage of S-phase cells in the SNP+ group decreased slightly at 8 h, and it further declined at 12 h (*p* < 0.001). In sync with the latter trend, at 12 h, there was a commensurate rise in the percentage of cells in the G_0_/G_1_ and G_2_/M phases (*p* < 0.01). At 24 h, in the SNP+ cell group, we observed respectively a persistent reduction and increase in the size of the S-phase and G_0_/G_1_-phase subgroups (*p* < 0.001), while the size of the G_2_/M–phase subgroup returned to the baseline level. It is worth noting that, as compared to the SNP+ group, the SNP– group exhibited a less substantial cell-cycle perturbation trend over a 24 h period as shown in Fig. 4d.

### Upregulation of HSP-70 genes induced by ultrasound exposure in presence of microbubbles

The cell-cycle disruption trends observed in the SNP+ and SNP– cell groups indicated that their subcellular stress signal expressions may be concomitantly altered over time. To investigate the potential downstream molecular bioeffects of ultrasound exposure in the presence of microbubbles, we evaluated the post-exposure transcription levels of three HSP-70 genes (HSPA1A, HSPA1B and HSPA6) by quantitative polymerase chain reaction (qPCR) analysis. These three genes encode the key proteins of the HSP70 family, which play a cytoprotective role in a cell’s protein folding machinery and is highly conserved in all organisms.

**Figure 5:**
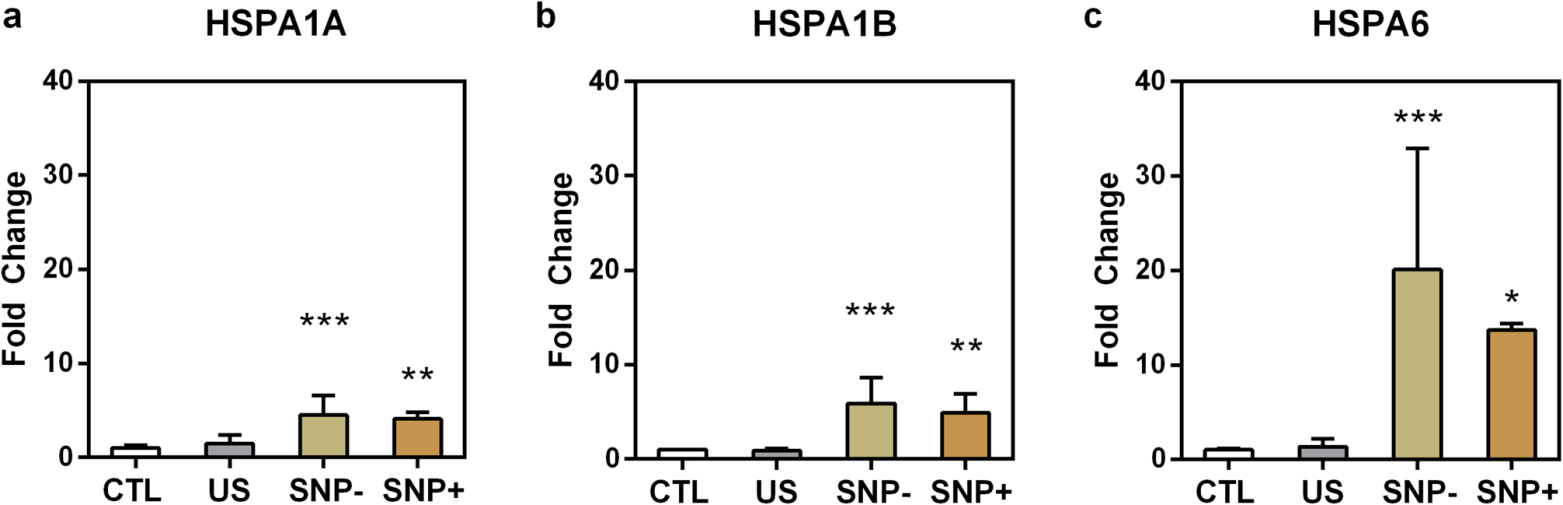
Messenger ribonucleic acid (mRNA) expressions of three genes in the heat shock protein-70 (HSP-70) family for four sorted cell groups (SNP+, SNP–, US, CTL) at 3h after exposure. Fold changes relative to the CTL group are shown for: **(a)** HSPA1A; **(b)** HSPA1B; **(c)** HSPA6. Error bars denote standard deviation (*N*=6) (*, **, and *** respectively denote *p *< 0.05, *p *< 0.01, and *p *< 0.001 versus CTL group).

Figure 5 shows the messenger ribonucleic acid (mRNA) expression level of the HSPA1A, HSPA1B and HSPA6 genes at 3 h after exposure. For SNP+ cells, the expression of HSPA1A, HSPA1B, and HSPA6 were found to be increased by 4.1-fold (*p* < 0.001), 4.9-fold (*p* < 0.01), and 13.7-fold (*p* < 0.05) respectively on average. A similar HSP70-related gene upregulation trend was observed in the SNP– cell group. In particular, the HSPA1A, HSPA1B and HSPA6 genes of SNP– cells were upregulated by 4.5-fold (*p* < 0.001), 5.9-fold (*p* < 0.001), and 20.1-fold (*p* < 0.001) respectively on average. Between the SNP+ and SNP– cell groups, no statistically significant difference was found in their HSP-70 gene upregulation trends. These results should be contrasted with that for the US cell group, which did not exhibit statistically significant changes in the three genes’ expression level.

## Discussion

### Integrative perspective of experimental results

 Sonoporation, despite being considered as a promising membrane perforation technique with strong application potential in drug/gene delivery, has not yet reached widespread clinical use largely because its mechanistic foundations are still not well characterized. Results from our investigation have allowed us to form new perspectives on the cellular impact of sonoporation by revealing how this membrane perforation approach, even whilst it may not affect the viability of target cells, may jeopardize post-exposure cellular homeostasis over a prolonged, 24 h period. Our new experimental insights were uniquely derived from the use of a rigorous experimental protocol that allowed viable cells with confirmed occurrence of sonoporation (and its absence) to be isolated for group-specific bioassay analysis (Fig. 1). Accordingly, our findings are seemingly more specific than those reported in past studies that did not account for the heterogeneity of bioeffects that tend to emerge in a sonicated cell population.

**Figure 6:**
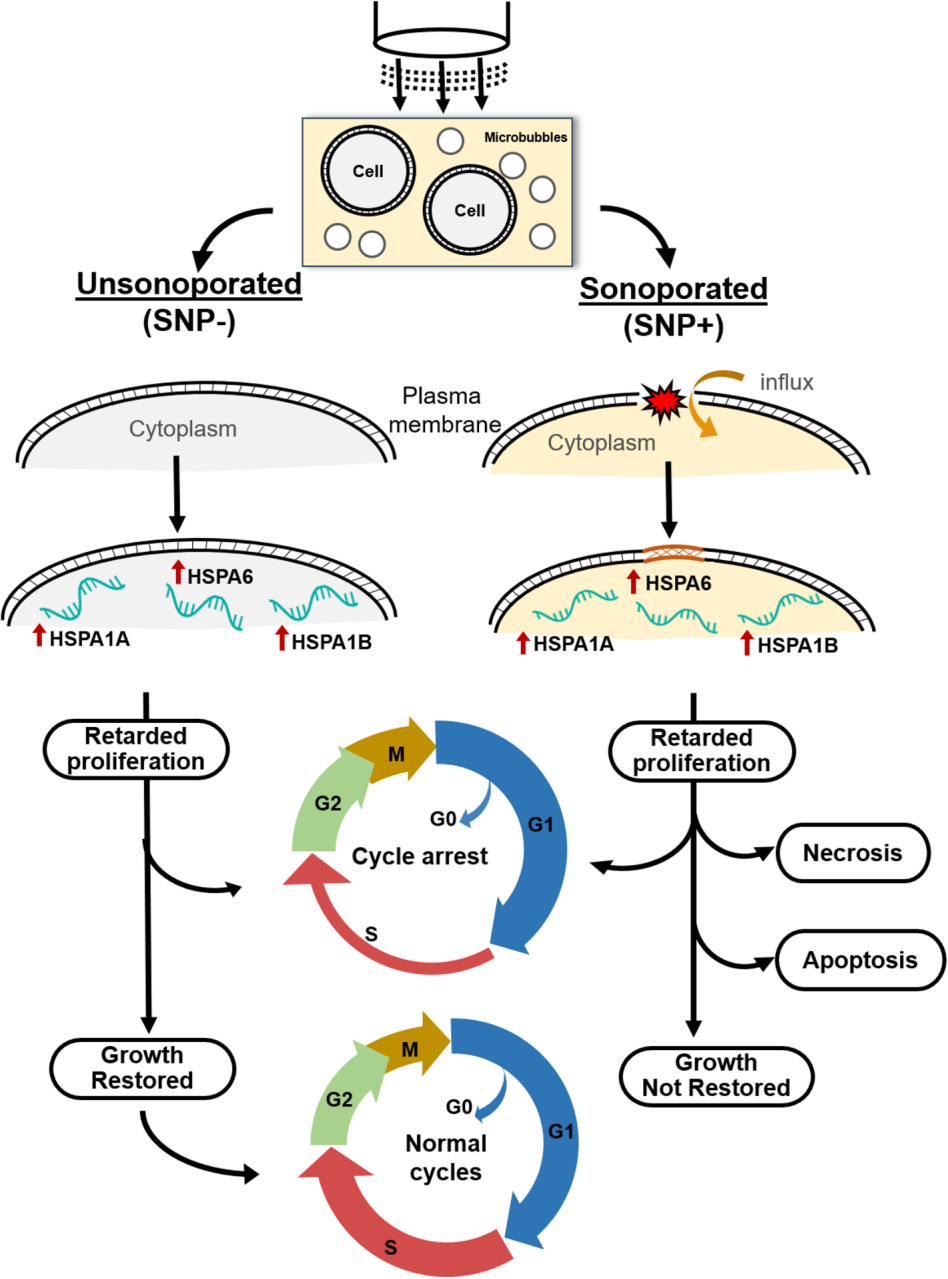
Downstream cellular impact observed in unsonoporated cells (left) and sonoporated cells (right), as derived from the experimental findings. Both cell groups experienced upregulation of HSP-70 expression level and disruption of cell-cycle distribution. Unsonoporated cells eventually restored their proliferation trend (after 24 h), whereas many sonoporated cells failed to recover and became apoptotic or necrotic.

The primary findings of this investigation are summarized in Fig. 6. In general, our study has disentangled the downstream cellular impact of viable, sonoporated cells (the SNP+ group) from that of unsonoporated ones (the SNP– group). Over a 3-h time frame, sonoporated cells exhibited an upregulation of their HSP-70 expression levels (Fig. 5; gold bars). In the face of proteotoxic stress, such an enhanced expression of HSP-70 is supposed to play a cytoprotective role in a stressed cell’s quest to maintain viability and to facilitate protein damage repair^30^. Nonetheless, our data has shown that a significant fraction of sonoporated cells lost viability over time as they entered apoptosis and necrosis (Fig. 2; gold boxes). In turn, the proliferation trend of sonoporated cells was interrupted over a 24-h period (Fig. 3; orange line). The correpsonding cell-cycle distribution also became disordered (Fig. 4e), wherein many sonoporated cells, after 12 h, had failed to progress into DNA synthesis (Fig. 4b; gold bars) and had stalled from undergoing mitosis (Fig. 4c; gold bars). Taken together, these findings have demonstrated that, in sonoporated cells, cytoprotective bioeffects may be triggered within the first few hours after exposure as these cells attempt to restore homeostasis after membrane resealing; yet, many of them failed to recover and ultimately lost viability over a 24 h period.

### Comparison of bioeffects between sonicated cell groups

As illustrated in Fig. 6, the fate of sonoporated cells is generally different from that observed in viable, unsonoporated cells (i.e. cells without membrane disruption after being subjected to ultrasound exposure in the presence of microbubbles), even though these two cell groups have exhibited similar cytoprotective behavior. Over a 24-h period, unsonoporated cells mostly remained viable (Fig. 2; dark gray boxes) and showed proliferation characteristics (Fig. 3; gold line). Also, they had an increase in HSP-70 expression 3 h after exposure (Fig. 5; yellow bars) and had a perturbed cell-cycle distribution by 12 h after exposure (Fig. 4d). These trends altogether suggest that unsonoporated cells, despite not undergoing membrane perforation, may still be reversibly stressed by ultrasound exposure in the presence of microbubbles, but they were able to cope with this stress without losing viability over time.

Regarding the downstream behavior of unsonoporated cells, one plausible mechanistic inference that can be drawn is that, because they were mechanically perturbed by the cavitational interactions between ultrasound and microbubbles, they consequently activated their cytoprotective signaling pathways and temporarily slowed their cell-cycle dynamics to promote cellular recovery. After they have restored cellular homeostasis, unsonoporated cells would resume their normal proliferation trend. Such a time-varying biological response is seemingly attributed to the presence of microbubbles during ultrasound exposure.

In contrast, for cells that were sonicated in the absence of microbubbles (the US group), they did not exhibit significant changes in viability (Fig. 2; light gray boxes), cell-cycle distribution (Fig. 4a–c; gray bars), and HSP-70 expression levels (Fig. 5; gray bars) as compared to the sham exposure group (the CTL group). Interestingly, the US group exhibited a growth spurt in its proliferation trend (Fig. 3; gray line) that resulted in a larger post-exposure population size than the CTL group (Fig. 3; black line). This result corroborates with previous understanding that short-term cytomechanical perturbations with low-intensity ultrasound may lead to long-term growth stimulation of cells^31^. In general, our experiments have demonstrated that the downstream behavior of sonoporated and unsonoporated cells is characteristically different from that for cells that were sonicated in the absence of microbubbles (i.e. the US group in the presented results).

### Significance of research findings

Overall, our investigation has unveiled that multifaceted bioeffects, including both cytoprotective and cytotoxic phenomena, may be triggered on a time-lapsed basis after a sonoporation episode. This work has served well to prompt the need to re-consider how sonoporation should be more rationally incorporated in a drug delivery paradigm to enhance its efficacy. After all, in therapeutic studies, long-term cell viability is an essential efficacy indicator^32, 33^, and it plays a major role in cancer therapy in determining chemotherapy dosage and in planning treatment cycles^34, 35^. It would thus be worthwhile to devise a refined sonoporation-mediated drug delivery protocol to exploit the observed bioeffects to our advantage. For instance, one may leverage the cell-cycle disruption trends observed in this work to develop a revised treatment protocol that involves the use of cycle-specific drugs^36^.

Regarding post-sonoporation cytoprotective maneuvers, we have identified the potential molecular facilitators involved in this process. In particular, HSP-70 genes were studied as the molecular targets. Interestingly, both sonoporated and unsonoporated cells exhibited a significant upregulation of HSP-70 genes a few hours after sonication in the presence of microbubbles. This finding suggested that these cells’ self-protection, pro-survival machinery had been activated in response to the exposure. It is worth reiterating that, while unsonoporated cells managed to restore homeostasis within 24 h, a significant fraction of sonoporated cells had failed to recover and restore normal cell division activities. This difference in the time-lapsed developmental behavior of the two sonicated cell subpopulations indicate that other molecular pathways in addition to HSP-70 are likely involved at different post-exposure time points. In the future, it would be worthwhile to establish a more detailed account of the molecular orchestration of sonoporated cell fate (and compare them to that for unsonoporated cells). Further investigation on this topic will deepen our mechanistic understanding on sonoporation-induced bioeffects.

It is worth emphasizing that our experiment protocol was specifically designed to analyze the downstream behavior of viable, sonoporated cells among a sonicated cell population. The ultrasound-microbubble mediated exposure condition used in our protocol was well defined in vitro and was kept constant. Specifically, cells and microbubbles within the cell chamber were held in suspension such that they were uniformly distributed to ensure that cells were consistently sonicated with 1 MHz ultrasound pulses (with 0.50 MPa in-situ peak negative pressure) in the presence of microbubbles (with 1:1 cell-to-bubble ratio). This way of realizing sonoporation is naturally more meticulous, yet different, from that for in vivo applications, where microbubbles are injected as a bolus into the blood stream and the in situ ultrasound-microbubble mediated exposure conditions are not characterized. Since HL-60 leukemia cells are after all circulating cells in the vasculature, one further investigation that can be performed in the future will be to examine the extent of the observed sonoporation bioeffects in a flow phantom environment that more closely resembles physiologically relevant ultrasound-microbubble mediated exposure conditions^37^. This further study will help to connect our reported research findings with in vivo application settings.

## Conclusion

Sonoporation is still in need of refining its efficiency such that its therapeutic efficacy can be critically enhanced. Our investigation has served well to underscore how sonoporation may instigate a variety of cellular impact over time. This new body of mechanistic knowledge can be leveraged in different ways. For instance, in sonoporation-mediated chemotherapy, where the ultimate outcome is cell death, the intended drug action can be designed to synergize with the antiproliferation impact induced by sonoporation. In other drug delivery applications where cell survival is desired, efficiency may be improved by targeting HSP-70 signaling as a potential modulation pathway to boost the cytoprotective response of sonoporated cells, since our investigation has revealed that HSP-70 genes were upregulated as a cytoprotective maneuver to promote post-sonoporation cell recovery. In general, realizing rational use of sonoporation-mediated drug delivery will significantly broaden its application potential in biomedicine.

## Materials and methods

### Sonication protocol

#### Exposure platform

The ultrasound apparatus employed in this investigation was an immersion-based exposure setup that coupled a secure-sealed cell chamber to a mounted ultrasound transducer with calibrated field levels within a 37 °C degassed water bath. Its design details have been established in our previous work^27^. In brief, its hardware components included a cylindrical ultrasound transducer with 1 MHz center frequency (Ø25mm, Advanced Devices, Wakefield, MA, USA), an arbitrary waveform generator (33120A; Agilent Technologies, Santa Clara, CA, USA), a broadband amplifier with 50 dB gain (2100L; E&I, Rochester, NY, USA), and a three-level acrylic-based mounting platform. The transducer and the secure-sealed cell chamber were respectively mounted to the bottom and middle panels of the mounting platform, and they were distanced at 10 cm apart. The platform’s top panel served as an acoustic shield that prevented acoustic standing waves from emerging in the cell chamber.

#### Acoustic parameters

During operation, sinusoidal pulses were first generated by the exposure platform’s waveform generator, and they were fed into the broadband amplifier that relayed the amplified output to the ultrasound transducer. The waveform frequency was set at 1 MHz in accordance with the transducer’s center frequency. Also, the pulse repetition frequency was set to 1 kHz, and the duration of each pulse was 100 cycles. These parameters corresponded to a 10% duty cycle. Exposure duration was set to 30 s in each experiment run. As calibrated previously^27^, in situ ultrasound exposure level was measured with a needle hydrophone (HMB-0500; Onda Corporation, Sunnyvale, CA, USA) and an automated three-axis micro-positioner (ASTS-01; Onda Corporation). The spatial-average peak negative pressure was measured to be 0.50 MPa in the cell chamber region. The corresponding intensity values of interest were 9.71 W/cm^2^ (spatial-peak, pulse-average) and 0.97 W/cm^2^ (spatial-peak, time-average), and the delivered acoustic energy density was 29.1 J/cm^2^.

### Sonicated cell sample preparation

#### Cell culture

HL-60 leukemia cells (CCL-240; American Type Culture Collection, Manassas, VA, USA) were chosen as the experimental cell line in this study. Owing to its intrinsic deficiency in pro-apoptotic regulatory proteins^34^, this immortal cell line was naturally more resistant against pro-apoptotic stress. Such property served well as an intrinsic control in our investigation of anti-proliferation effects^35^. The HL-60 cells were routinely cultured in Roswell Park Memorial Institute (RPMI) 1640 medium (R8758; Sigma-Aldrich, St. Louis, MO, USA) and were supplemented with 10% fetal bovine serum (American Type Culture Collection). The cell culture incubator was kept at 37 °C with 5% carbon dioxide (CO_2_). Cell density were constantly monitored to ensure that they did not exceed 10^6^ cells/ml. As such, exponential cell growth can be maintained. HL-60 cells were sub-cultured every three days. During sub-culture, cells were harvested in a sterilized 15 ml centrifuge tube (BDAA352196; BD Biosciences, East Rutherford, NJ, USA) and were centrifuged at 1000 rpm for 5 min. Afterward, supernatants were removed and the harvested cells were resuspended with fresh RPMI medium.

#### Addition of sonoporation tracer

Calcein (C481, Invitrogen, Carlsbad, CA, USA) was employed as the exogenous, fluorescent sonoporation tracer to facilitate the sorting of sonoporated cells using flow cytometry. They were added to the cell suspension at a 10 µM concentration 10 min before cells were transferred to the cell chamber. Note that calcein (Ex: 495 nm/Em: 515 nm) is a 623 Da macromolecule with a radius of 0.65 nm, which is normally unable to permeate the cell membrane and remain extracellularly in the cell medium. Upon the occurrence of sonoporation, calcein would infiltrate the cell membrane via the perforated site and subsequently bind to cytoplasmic calcium ions, thereby resulting in the emission of green fluorescence from the cytoplasm of sonoporated cells. Hence, in our experiments, post-exposure intracellular presence of calcein fluorescence served well to indicate that sonoporation had occurred in that sonicated cell.

#### Addition of microbubbles

Commercial microbubbles (USphere Prime; TRUST Bio-Sonics, Taiwan) were used as the artificial gas nuclei for acoustic cavitation to trigger sonoporation. These microbubbles comprised a perfluorobutane gas core that was encapsulated by a phospholipid shell. They had a mean diameter of 2.09 µm, and less than 1.7% of the population were larger than 5 µm according to the manufacturer’s data. During experiments, microbubbles were added to the cell suspension 3 min before the ultrasound exposure at a cell-to-bubble ratio of 1:1. Note that the choice of cell-to-bubble ratio plays a significant role in the resulting sonoporation efficiency^20^. The 1:1 cell-to-bubble ratio was empirically found to provide a balance between inducing substantial sonoporation (> 30%) and minimizing instant cell death (< 20%) in our previous studies^25, 27^. With this cell-to-bubble ratio and the prescribed acoustic exposure parameters, over 99% of the supplemented microbubbles were found to have collapsed after the 30 s exposure, as observed under bright-field microscope.

#### Delivery of ultrasound exposure

Before exposure, the cell suspension (supplemented with microbubbles and calcein) was filled into the ultrasound exposure platform’s cell chamber, which was acoustically transparent and was sterilized beforehand^27^. The cell chamber was securely sealed after filling and was promptly placed at the middle panel of the exposure platform. Thereafter, ultrasound exposure was delivered as described in the previous subsection. After exposure, cells were immediately taken out from the cell chamber and were incubated for 5 min to allow sonoporated cells to reseal their perforation sites and, in turn, to restore the resistance against the uptake of exogeneous molecules. Note that the post-exposure incubation time was set based on previously published findings that showed membrane resealing to be typically taking place within 1 min after the onset of a resealable sonoporation episode^11^.

### Isolation of sonoporated cells

#### Labeling of non-viable cells

Following the post-exposure incubation period, sonicated cells were transferred to 5-ml sterile flow cytometry tubes (352054; BD Biosciences). After rinsing them twice with RPMI medium, they were stained for 15-min with 0.75 µM PI (P4170, Sigma-Aldrich) to label non-viable sonicated cells whose membrane remain compromised after the sonoporation episode and the 5 min post-exposure incubation period. Afterwards, cells were washed twice with PBS.

#### Identification of cell subpopulations with flow cytometry

Identification of subpopulations was achieved through calcein/PI bivariate fluorescence analysis of the sonicated cell samples using a flow cytometer (Aria III, BD Biosciences). In each flow cytometry run, data for 10^4^ cells per sample was acquired at a rate of 300 events/s. Also, calcein fluorescence was measured with a 530/30 nm filter, and PI fluorescence was detected with a 616/23 nm filter. Cells showing positive PI fluorescence were deemed to belong to the non-viable population, while those showing positive calcein fluorescence were deemed to belong to the sonoporated cell population. Accordingly, the bivariate flow cytometry analysis allowed us to identify subpopulations of viable sonoporated cells (PI–/calcein +), viable unsonoprated cells (PI–/calcein–), and non-viable cells that may or may not be sonoporated (PI + /calcein + and PI + /calcein–). Our previous data showed that among a sonicated HL-60 cell population, 41.9% were viable sonoporated cells^27^.

#### Cell sorting

Using the FACS functionality of the flow cytometer, viable sonoporated cells (SNP+) and viable unsonoporated cells (SNP–) were isolated by setting each subpopulation’s gate fluorescence level according to the observed bivariate fluorescence distribution in the post-exposure cell population sample. Non-viable cells were excluded from this sorting process. From each sorting experiment, 5 × 10^6^ cells for each targeted subpopulation were collected. Note that cells were sorted at a rate of 3000 events/s flow rate to ensure the targeted number of cells can be harvested efficiently within 30 min. During the cell sorting process, the sheath fluid of flow cytometer and the sample container were maintained at 4 °C to preserve cell viability^38^. The harvested cell populations were immediately re-incubated until the corresponding time points for conducting biological assays. Note that cells of the sham control group (CTL) and a sonicated cell group that received ultrasound exposure in the absence of microbubbles (US) also went through the identical cell sorting procedures.

### Biological assays

#### Apoptosis and necrosis analysis

Apoptosis and necrosis were analyzed using the Annexin-V/PI bivariate fluorescence assay at 3 h, 8 h, 12 h and 24 h after exposure according to the established protocol^39^. For each sorted cell sample, 2 × 10^5^ cells were harvested and were washed with 4 °C PBS. The samples were then resuspended in binding buffer (1 × HEPES buffer solution). After centrifugation and removal of supernatants, each cell population sample was incubated with 5 μl Annexin-V (A23202, Alexa Fluor 350 conjugate, Invitrogen) and 1 μl of 100 μg/mL PI in a 100 μl binding buffer at room temperature for 15 min. Annexin-V binding served to label instances of phosphatidylserine externalization manifested in cells undergoing early apoptosis, whereas cytoplasmic presence of PI served to label cells with compromised membrane permeability that is well regarded as a shared feature of late apoptotic and necrotic cells^39^. Subsequently, 400 μl binding buffer was added to each sample on ice. Flow cytometry was then applied using fluorescence with Ex/Em of 346/440 nm laser for Annexin-V and Ex/Em of 488/625 nm for PI. The cell group was then displayed as scatter plots with horizontal and vertical axis representing Annexin-V and PI level respectively. From this analysis, three groups of cells could be identified: (a) early apoptotic cells (PI–/Annexin V +); (b) late apoptotic or necrotic cells (PI +); (c) viable cells (PI–/annexin-V–). Note that this protocol cannot identify dead cells that had already been lysed at a given post-exposure time as they were inevitably removed by centrifugation.

#### Cell proliferation analysis

The CCK-8 colorimetric assay (Dojindo Molecular Technologies, Inc., MD, USA) was carried out to determine the number of viable cells at different post-exposure times. This assay was chosen in view of its low cytotoxicity to living cells and high sensitivity comparing with other proliferation assays such as MTT. In each experiment, a sorted cell subpopulation sample was centrifuged at 1000 rpm for 5 min at 37 °C and was resuspended into the culture medium at a density of 2 × 10^5^ cells/ml. The cell suspension was then transferred to a 96-well plate with 100 µl per well. Subsequently, 10 µl of CCK-8 solution was added to each well. The 96-well place was incubated for 2 h at 37 °C and 5% CO_2_ to foster reaction. The plate was afterward transferred to a spectrophotometer (Multiskan Go; Thermo Scientific Inc.) and the absorbance at 450 nm was measured. In each experiment, calibration samples were prepared by inoculating 5 × 10^3^, 1 × 10^4^, 2 × 10^4^, 4 × 10^4^ cells/well respectively with identical volume of 100 µl.

#### Cell cycle analysis

The cell-cycle distribution at different time points was examined by fixing the cell subpopulation sample and analyzing the fixed cells’ DNA contents via PI fluorescence staining and flow cytometry. The sorted cell populations were extracted at 3 h, 8 h, 12 h and 24 h after exposure and was fixed with 75% ice-cold ethanol. The fixed samples were stored at –20 °C for further analysis. Before carrying out the cell-cycle analysis, the fixed samples were washed with PBS to remove the ethanol. Subsequently, the samples were stained with 0.5 ml of PI solution (75 mM) in the dark at room temperature. The fluorescence of the PI-stained cells was analyzed by a flow cytometer following the standard protocol^40^. Histograms of PI fluorescence, which represent distribution of cellular DNA contents instead of cell viability because cells were already fixed, were then formed from 10,000 cell counts for each sample. Cells at G_0_/G_1_-,S- and G_2_/M-phases were gated and quantified from the DNA histogram.

#### HSP-70 expression analysis

qPCR analysis was conducted to evaluate the mRNA level of HSPA6, HASPA1A, HSPA1B in each sorted population at 3 h after exposure. The three genes were chosen as they encode the 70-kDa heat shock proteins (HSP70) that serve a cytoprotective function in response to disturbance of cell homeostasis via chaperon regulation^26^. Prior to the analysis, 1 × 10^7^ cells were harvested for each sample and the total RNA of the sample was isolated with the standard TRIzol (Invitrogen) method. To verify the quality of the extracted RNA, a quantitative estimation was conducted with the Multiskan GO spectrophotometer, and its integrity was double confirmed by agarose gel electrophoresis. cDNA was synthesized from 100 ng of total RNA using the TaqMan reverse transcription kit (Thermofisher) according to the manufacturer’s protocol. The qPCR reaction mix was prepared using a SYBR Fast qPCR Kit (Kapa Biosystems). Cycling and gene expression detection was conducted with a real-time PCR system (ABI-7500; Applied Biosystems). The primers used in the experiments are listed in Table 2. RNA quantity was normalized using GAPDH content, and gene expression was quantified according to the 2^-ΔΔCT^ method^41^.

**Table 2:** List of primer sequences used in this investigation.

Gene	Oligo	Sequence
*HSPA6*	Forward	*GATGTGTCGGTTCTCTCCATTG*
	Reverse	*CTTCCATGAAGTGGTTCACGA*
*HSPAJA*	Forward	*CCGAGAAGGACGAGTTTGAG*
	Reverse	*ACAAAAACAGCAATCTTGGAAAGG*
*HSPAJB*	Forward	*TGAAGCAGCAAAGAGCTGAA*
	Reverse	*GTGGATTAGGGGCCTTTGTT*
*DNAJBJ*	Forward	*TTCCCCAGACATCAAGAACC*
	Reverse	*ACCCTCTCATGGTCCACAAC*
*GAPDH*	Forward Reverse	*CATCAATGGAAATCCCATCA TTCTCCATGGTGGTGAAGAC*

#### Statistical evaluation

For all conducted bioassays, the analysis of variance (ANOVA) test was applied to the acquired measurements to evaluate whether there were statistically significant differences in the data samples between cell groups and between different post-exposure time points. This statistical evaluation task was conducted using OriginPro (ver. 9.0; OriginLab Corporation, Northampton, MA, USA). Trends of statistical significance were identified as ones with *p* values of < 0.05, < 0.01, and < 0.001, which respectively correspond to confidence intervals of 95%, 99%, and 99.9%.

## References

[1] Ibsen S, Schutt CE, Esener S (2013). Microbubble-mediated ultrasound therapy: A review of its potential in cancer treatment. Drug Des. Dev. Ther..

[2] Qin, J., Wang, T.-Y., & Willmann, J. K. Sonoporation: Applications for cancer therapy. In: *Therapeutic Ultrasound* 263–291 (Springer, 2016). 10.1007/978-3-319-22536-4_15 26486343

[3] Delalande A, Kotopoulis S, Postema M, Midoux P, Pichon C (2013). Sonoporation: Mechanistic insights and ongoing challenges for gene transfer. Gene.

[4] Bouakaz, A., Zeghimi, A., & Doinikov, A. A. Sonoporation: Concept and mechanisms. in *Therapeutic Ultrasound *175–189. (Springer, 2016). 10.1007/978-3-319-22536-4_10 26486338

[5] Mignet, N., Marie, C., Delalande, A., Manta, S., Bureau, M.-F., Renault, G., Scherman, D., & Pichon, C. Microbubbles for nucleic acid delivery in liver using mild sonoporation. in *Nanotechnology for Nucleic Acid Delivery *377–387 (Springer, 2019). 10.1007/978-1-4939-9092-4_25 30838630

[6] Nejad SM, Hosseini H, Akiyama H, Tachibana K (2016). Reparable cell sonoporation in suspension: Theranostic potential of microbubble. Theranostics.

[7] Kudo N, Okada K, Yamamoto K (2009). Sonoporation by single-shot pulsed ultrasound with microbubbles adjacent to cells. Biophys. J ..

[8] Liu Y, Yan J, Prausnitz MR (2012). Can ultrasound enable efficient intracellular uptake of molecules? A retrospective literature review and analysis. Ultrasound Med. Biol..

[9] Campbell P, Prausnitz MR (2007). Future directions for therapeutic ultrasound. Ultrasound Med. Biol..

[10] Lentacker I, de Cock I, Deckers R, de Smedt SC, Moonen CTW (2014). Understanding ultrasound induced sonoporation: Definitions and underlying mechanisms. Adv. Drug Deliv. Rev..

[11] Hu Y, Wan JMF, Yu ACH (2013). Membrane perforation and recovery dynamics in microbubble-mediated sonoporation. Ultrasound Med. Biol..

[12] Helfield B, Chen X, Watkins SC, Villaneuva FS (2016). Biophysical insight into mechanisms of sonoporation. Proc. Natl. Acad. Sci..

[13] Chen X, Leow RS, Hu Y, Wan JMF, Yu ACH (2014). Single-site sonoporation disrupts actin cytoskeleton organization. J. R. Soc. Interface.

[14] Beekers I (2020). High-resolution imaging of intracellular calcium fluctuations by oscillating microbubbles. Ultrasound Med. Biol..

[15] Leow RS, Wan JMF, Yu ACH (2015). Membrane blebbing as a recovery manoeuvre in site-specific sonoporation mediated by targeted microbubbles. J. R. Soc. Interface.

[16] Qin P, Han T, Yu ACH, Xu L (2018). Mechanistic understanding the bioeffects of ultrasound-driven microbubbles to enhance macromolecule delivery. J. Control. Release.

[17] Guzman, H. R., Nguyen, D. X., Khan, S., & Prausnitz, M. R. Ultrasound-mediated disruption of cell membranes. II. Heterogeneous effects on cells. *J. Acoust. Soc. Am.* **110**, 597–606 (2001). 10.1121/1.1376130 11508985

[18] Qin P, Xu L, Han T, Du L, Yu ACH (2016). Effect of non-acoustic parameters on heterogeneous sonoporation mediated by single-pulse ultrasound and microbubbles. Ultrason. Sonochem..

[19] Hutcheson JD, Schlicher RK, Hicks HK, Prausnitz MR (2010). Saving cells from ultrasound-induced apoptosis: Quantification of cell death and uptake following sonication and effects of targeted calcium chelation. Ultrasound Med. Biol..

[20] Guzmán HR, McNamara AJ, Nguyen DX, Prausnitz MR (2003). Bioeffects caused by changes in acoustic cavitation bubble density and cell concentration: A unified explanation based on cell-to-bubble ratio and blast radius. Ultrasound Med. Biol..

[21] Feril LB, Kondo T (2004). Biological effects of low intensity ultrasound: the mechanism involved, and its implications on therapy and on biosafety of ultrasound. J. Radiat. Res..

[22] Miller DL, Dou C (2009). Induction of apoptosis in sonoporation and ultrasonic gene transfer. Ultrasound Med. Biol..

[23] Zhong W, Sit WH, Wan JMF, Yu ACH (2011). Sonoporation induces apoptosis and cell cycle arrest in human promyelocytic leukemia cells. Ultrasound Med. Biol..

[24] Zhang J-Z, Saggar JK, Zhou Z-L, Hu B (2012). Different effects of sonoporation on cell morphology and viability. Bosnian J. Basic Med. Sci..

[25] Chen X, Wan JMF, Yu ACH (2013). Sonoporation as a cellular stress: Induction of morphological repression and developmental delays. Ultrasound Med. Biol..

[26] Mayer MP, Bukau B (2005). Hsp70 chaperones: Cellular functions and molecular mechanism. Cell. Mol. Life Sci..

[27] Duan X, Yu ACH, Wan JMF (2019). Cellular bioeffect investigations on low-intensity pulsed ultrasound and sonoporation: Platform design and flow cytometry protocol. IEEE Trans. Ultrason. Ferroelectr. Freq. Control.

[28] Wolf D, Rotter V (1985). Major deletions in the gene encoding the p53 tumor antigen cause lack of p53 expression in HL-60 cells. Proc. Natl. Acad. Sci..

[29] Zhong W, Chen X, Jiang P, Wan JMF, Qin P, Yu ACH (2013). Induction of endoplasmic reticulum stress by sonoporation: Linkage to mitochondria-mediated apoptosis initiation. Ultrasound Med. Biol..

[30] Radons J (2016). The human HSP70 family of chaperones: Where do we stand?. Cell Stress Chaperones.

[31] Hu Y, Wan JMF, Yu ACH (2014). Cytomechanical perturbations during low-intensity ultrasound pulsing. Ultrasound Med. Biol..

[32] Crescenzi E, Varriale L, Iovino M, Chiaviello A, Veneziani BM, Palumbo G (2004). Photodynamic therapy with indocyanine green complements and enhances low-dose cisplatin cytotoxicity in MCF-7 breast cancer cells. Mol. Cancer Ther..

[33] Kirby TO (2004). A novel ex vivo model system for evaluation of conditionally replicative adenoviruses therapeutic efficacy and toxicity. Clin. Cancer Res..

[34] Naidu M (2004). Chemotherapy-induced and/or radiation therapy-induced oral mucositis—Complicating the treatment of cancer. Neoplasia.

[35] Chugh, R. *et al*. A preclinical evaluation of Minnelide as a therapeutic agent against pancreatic cancer. *Sci. Transl. Med*. **4**, 156ra39 (2012). 10.1126/scitranslmed.3004334 PMC3656604 23076356

[36] Varghese L, Agarwal C, Tyagi A, Singh RP, Agarwal R (2005). Silibinin efficacy against human hepatocellular carcinoma. Clin. Cancer Res..

[37] Cheng M, Li F, Han T, Yu ACH, Qin P (2019). Effects of ultrasound pulse parameters on cavitation properties of flowing microbubbles under physiologically relevant conditions. Ultrason. Sonochem..

[38] Mocé-Llivina L, Jofre J (2004). A method to maintain mammalian cells for days alive at 4 C. Cytotechnology.

[39] Vermes I, Haanen C, Steffens-Nakken H, Reutellingsperger C (1995). A novel assay for apoptosis flow cytometric detection of phosphatidylserine expression on early apoptotic cells using fluorescein labelled annexin V. J. Immunol. Methods.

[40] Nunez R (2001). DNA measurement and cell cycle analysis by flow cytometry. Curr. Issues Mol. Biol..

[41] Livak KJ, Schmittgen TD (2001). Analysis of relative gene expression data using real-time quantitative PCR and the 2^−ΔΔCT^ method. Methods.

